# *In Vitro* and *In Vivo* Studies on Protective Action of *N*-Phenethyl Caffeamide against Photodamage of Skin

**DOI:** 10.1371/journal.pone.0136777

**Published:** 2015-09-14

**Authors:** Yueh-Hsiung Kuo, Chien-Wen Chen, Yin Chu, Ping Lin, Hsiu-Mei Chiang

**Affiliations:** 1 Department of Chinese Pharmaceutical Sciences and Chinese Medicine Resources, China Medical University, Taichung 404, Taiwan; 2 Department of Biotechnology, Asia University, Taichung 413, Taiwan; 3 Department of Cosmeceutics, China Medical University, Taichung 404, Taiwan; University of Alabama at Birmingham, UNITED STATES

## Abstract

In our previous study, *N*-phenethyl caffeamide (K36) was proved to act as an antioxidant and an antiphotoaging agent by inhibiting type I procollagen degradation and stimulating collagen synthesis in human skin fibroblasts. In the present study, *in vitro* and *in vivo* experiments were conducted to investigate the mechanism of action and the antiinflammatory and antiphotoaging activity of K36. K36 reduced UVB-induced cyclooxygenase-2 (COX-2) and inducible nitric oxide synthases (iNOS) expression by regulating IκB and *p*-IκB expression. K36 also inhibited the nuclear translocation of NF-κB. Furthermore, the inhibition of mitogen-activated protein (MAP) kinases by K36 was attributed to the downregulation of COX-2. Topically applying K36 led to efficient antiwrinkle formation and reduced UVB-induced erythema and thickness of epidermis in hairless mice. In addition, K36 penetrated into the skin of hairless mice. Our findings show that K36 has significant beneficial effects on antioxidant, antiinflammatory, and antiphotoaging activity and suggest that K36 can be developed as an antiaging agent for cosmetic and skin care products.

## Introduction

Overexposure to ultraviolet (UV) irradiation causes oxidative stress, inflammation, hyperpigmentation, mutation, and degradation of the extracellular matrix (ECM), resulting in wrinkling and skin cancer [1]. UV irradiation generates reactive oxygen species (ROS), triggering signal transduction cascades and activating inflammatory cytokines [2, 3]. These cytokines subsequently activate protein kinases, such as the mitogen-activated protein kinases (MAP kinases) and regulate downstream transcription factors [e.g., cyclooxygenase-2 (COX-2) and nuclear factor-κB (NF-κB)] causing an inflammatory response [4]. In addition, MAP kinases were shown to directly phosphorylate the AP-1 complex and NF-κB proteins, promoting the nuclear translocation of their transcription factors [5].

UVB irradiation increases the activity levels of phospholipase A_2_ and COX-2, resulting in inflammation, erythema, and related symptoms of the skin [6]. Furthermore, UV irradiation produces ROS to attack cell membrane lipids, increasing the prostaglandin E_2_ (PGE_2_) concentration and generating nitric oxide (NO) [7]. UVB irradiation activates transcription factors such as AP-1 and NF-κB [8]. NF-κB (p65/p50) is a transcription factor associated with inflammation that combines with IκB to form an inactive cytoplasmic complex. Cytokines and MAP kinases activate the IκB kinase, causing the ubiquitination of IκB, and NF-κB undergoes nuclear translocation to modulate the gene transcription and protein expression of iNOS and COX-2 [9].

Studies have reported that substances exhibiting antioxidant and antiinflammatory activity and inhibiting MMP expression can protect the skin from photodamage [10–12]. In addition, serotonin and melatonin synthesis in skin also play important roles in protection from photodamage and topical applied melatonin or its metabolites exhibit potent antioxidation to defence skin from UV-induced aging and carcinogenesis [12]. *N*-phenethyl caffeamide (K36) is an analogue of caffeic acid phenethyl ester (CAPE), an active component of propolis [13]. We previously reported that K36 exhibited antiphotodamage activity by scavenging intracellular ROS, inhibiting the MAPK/AP-1/MMP pathway, and alleviating UV-induced collagen decomposition in human skin fibroblasts [14]. An analogue of K36, (N-(4-bromophenyl) caffeamide, inhibited melanin biosynthesis in another study [15]. In addition, a study reported that the antioxidant and ROS-scavenging capabilities of CAPE accelerate the healing of full-thickness wounds [16]. In this study, we investigated the antiinflammatory activity of K36 in human skin fibroblasts as well as the prevention of photodamage in hairless mice. We also evaluated the percutaneous absorption of K36 after topical application to mice models to determine the feasibility of its clinical use.

## Materials and Methods

### Materials

JNK inhibitor II, PD98059, and SB203580 were purchased from Calbiochem (Darmstadt, Germany). Sodium dodecyl sulfate (SDS), Tris, and Tween 20 were obtained from the USB Corporation (Cleveland, OH, USA). Bovine serum albumin, dimethyl sulfoxide, leupeptin, paraformaldehyde, and phenylmethylsulfonyl fluoride were purchased from the Sigma-Aldrich Corporation (St. Louis, MO, USA). Dulbecco's modified Eagle's medium (DMEM), fetal bovine serum (FBS), penicillin-streptomycin, the ProLong Gold antifade reagent with 4',6-diamidino-2-phenylindole (DAPI), and trypsin-EDTA and were purchased from Gibco, Invitrogen (Carlsbad, CA, USA). The Bradford reagent was obtained from Bio-Rad Laboratories (Berkeley, CA, USA). The ECL Western blotting detection reagent and PageRuler prestained protein ladder were purchased from Amersham Biosciences (England). All other chemicals used in this study were of reagent grade.

### Cell culture

Human foreskin fibroblasts (Hs68) were purchased from the Bioresource Collection and Research Center (Hsinchu, Taiwan) and cultured in DMEM supplemented with 10% FBS, 100 U/mL of penicillin, and 100 U/mL of streptomycin at 37°C in a humidified atmosphere containing 5% CO_2_.

### UV irradiation

The cell culture medium was removed and cells were washed twice with PBS. The cells in PBS were irradiated with UVB at 40 mJ/cm^2^. UV irradiation was performed using a CL-1000M UV crosslinker (UVP, Upland, CA, USA). UVB was supplied by a closely spaced array of two UV lamps with a UV peak of 302 nm. The cells were subsequently incubated for the indicated time at 37°C in a humidified atmosphere of 5% CO_2_ in serum-free DMEM containing various concentrations of K36.

### Cytosolic or nuclear protein extraction

Cells were collected and homogenized in ice-cold PBS, and then centrifuged at 1500 rpm for 5 minutes at 4°C. The cells were resuspended in Buffer A (1 M HEPES, 1 M MgCl_2_, 1M KCl, 0.1 M dithiothreitol, 1M phenylmethylsulfonyl fluoride) containing protease inhibitor cocktails, and then incubated for 15 minutes on ice. NP-40 was added into the cells and vortexed for 10 seconds, and these homogenates were centrifuged at 14000 ×g for 5 minutes at 4°C to obtain cytosolic protein extracts. The pellets were washed twice in Buffer A and resuspended in iced lysis buffer for 30 minutes. The mixture was centrifuged at 14000 ×g for 10 min at 4°C to obtain nuclear protein extracts.

### Western blot analysis

The protein expression of K36 on UV-induced fibroblasts was measured using western blotting. The general procedures were followed as described previously [17–19]. Cells were collected, washed after the indicated treatments, and lysed. The total protein from these cells was separated on SDS-polyacrylamide gels by using electrophoresis and detected using specific antibodies. β-Actin was used to normalize the total protein amounts. The immobilized proteins were then transferred to polyvinylidene difluoride membranes and probed using antibodies. Immunoreactive proteins were detected using the ECL western blotting detection system (Fujifilm, LAS-4000, Japan) and the signal strengths were quantified using a densitometric program (MultiGauge V2.2).

### Immunofluorescence staining

The immunofluorescence staining for NF-κB was measured as previous study described [20]. The cells were cultivated on coverslips and incubated with various concentrations of K36 after UVB exposure. The cells were fixed with 4% paraformaldehyde and blocked with 5% nonfat milk containing 0.3% Triton X-100/PBS. After incubating with the primary antibody, the cells were incubated with the Alexa Fluor 488 antirabbit IgG secondary antibody (Invitrogen, USA). The unbound secondary antibody was removed using PBS. Thereafter, the samples were counterstained with the ProLong Gold antifade reagent with DAPI, and observed using a microscope (Leica DMIL, Germany). The visual scoring of the translocation on each slide was based on the characterization of 100 randomly selected cells.

### Experimental animals

BALB/cAnN.Cg-Foxn1nu/CrlNarl hairless female mice (5 weeks of age) were purchased from the National Laboratory Animal Center (Taipei, Taiwan). The mice were stabilized for 1 week prior to the study, housed in individual ventilated caging systems with a controlled temperature (24 ± 2°C), a controlled relative humidity (50 ± 10%), and 12 h light/dark cycles, and were provided a standard laboratory diet and water. All experimental protocols were approved by the Institutional Animal Use and Care Committee, China Medical University (protocol No. 100-124-N).

### UVB irradiation and topical application of K36

The mice were divided into control, UVB-irradiated, vehicle-treated and UVB-irradiated, UVB-irradiated and 25-μM-K36-treated, and UVB-irradiated and 100-μM-K36-treated groups. The UVB-irradiated mice were treated with 36 mJ/cm^2^ of UVB in the first week, 54 mJ/cm^2^ of UVB in the second to fourth weeks, 72 mJ/cm^2^ of UVB in the fifth to seventh weeks, and 108 mJ/cm^2^ of UVB in the eight to tenth weeks, three times per week [21]. The vehicle-treated UVB-irradiated mice were topically administered 50 μL of glycerol every day, and the K36-treated mice were topically administered equal volume of glycerol containing 25 μM or 100 μM K36 every day. The control mice remained untreated. All treatments were administered to the dorsal skin. The mice were sacrificed after the treatments, and the dorsal skin samples were excised and fixed in 10% formaldehyde. The skin slides were stained in hematoxylin and eosin or Masson’s trichrome, mounted with a coverslip, and examined under a microscope.

### *In vitro* transdermal delivery

Skin permeation by K36 was measured using a Franz vertical diffusion assembly with hairless mice modified according to a previous study [22]. The full-thickness dorsal skin of the female hairless mice was mounted between the donor and receptor compartments. The donor medium comprised 0.7 mL of 500 nmol K36 prepared in glycerol. The receptor medium comprised 30% ethanol in PBS (pH 7.4) and was used to maintain the sink condition of the permeants. The stirring rate was 600 rpm and the temperature was 37°C. The available diffusion area between the compartments was 0.95 cm^2^. After appropriate intervals, 0.3 mL of the receptor medium was withdrawn and replaced with an equal volume of the receptor medium. The contents of K36 in the receptor compartment were determined using HPLC-UV/VIS (Shimadzu, Kyoto, Japan). The cumulative amounts of K36 that permeated the skin were profiled as a function of time.

The K36 concentrations in the skin reservoir were determined after 48 h of transdermal delivery. The skin tissue was rinsed with water and blotted with paper to remove any residual sample on the skin surface. The tissue was weighed, minced using scissors, and placed in a blender (Bullet Blender, Next Advance, Inc., USA) containing 1 mL of methanol. The solution was centrifuged at 12,000 rpm for 5 min, and the supernatants were analyzed using HPLC-UV/VIS (Shimadzu, Kyoto, Japan) to determine the K36 concentrations.

### Data analysis

All measurements in the present study were obtained as the average of experiments performed in triplicate for independent experiments, and the results expressed as means ± standard deviation. The differences between the groups were analyzed to determine whether they were statistically significant by using the Student's *t* test or ANOVA. *P* < 0.05 was considered statistically significant. The dose–response relationship was analyzed using Jonckheere's trend test.

## Results

### Antiinflammatory effect of K36

**Fig 1:**
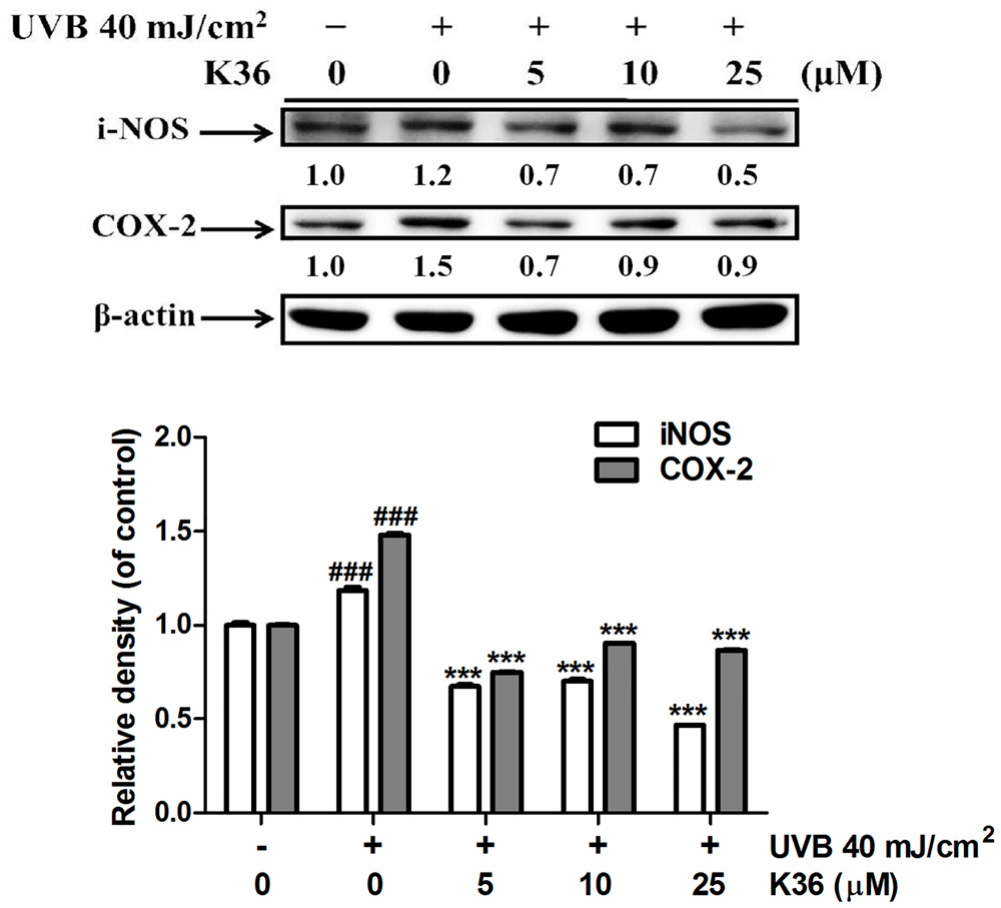
Effect of K36 on UVB-induced iNOS and COX-2 expression in human skin fibroblasts. Significant difference versus control group: ##, *P* < 0.01; ###, *P* < 0.001. Significant difference versus nontreatment group: **, *P* < 0.01; ***, *P* < 0.001.

**Fig 2:**
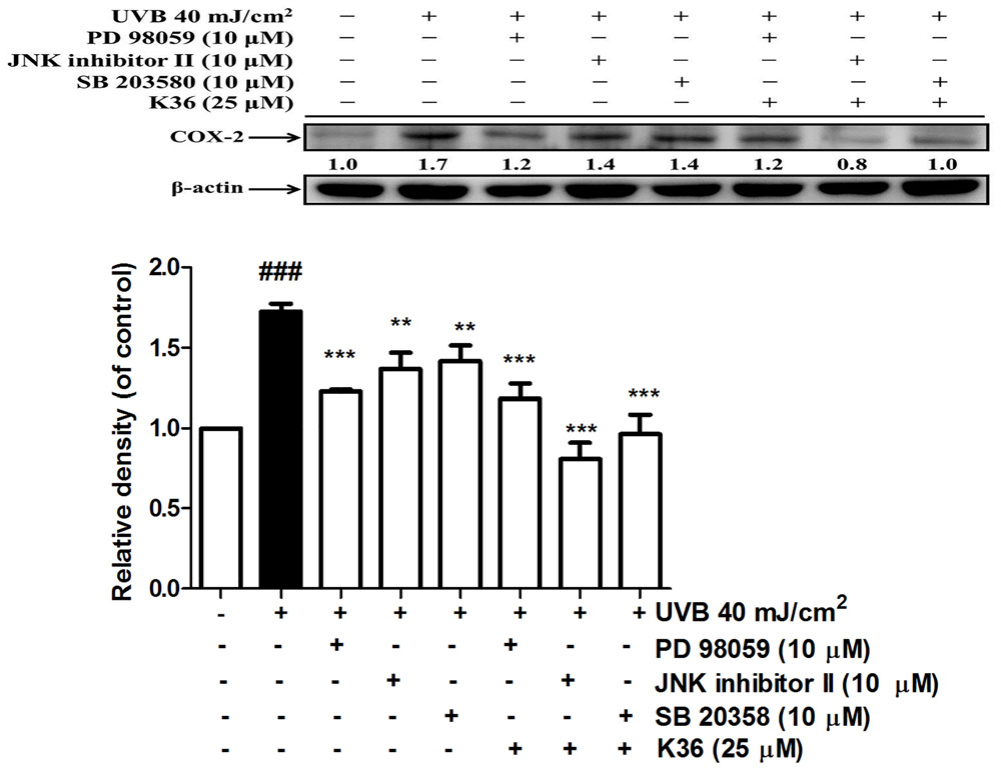
Effect of MAP kinase inhibitors and K36 on UVB-induced COX-2 expression in human skin fibroblasts. Significant difference versus control group: ###, *P* < 0.001. Significant difference versus nontreatment group: **, *P* < 0.01; ***, *P* < 0.001.

**Fig 3:**
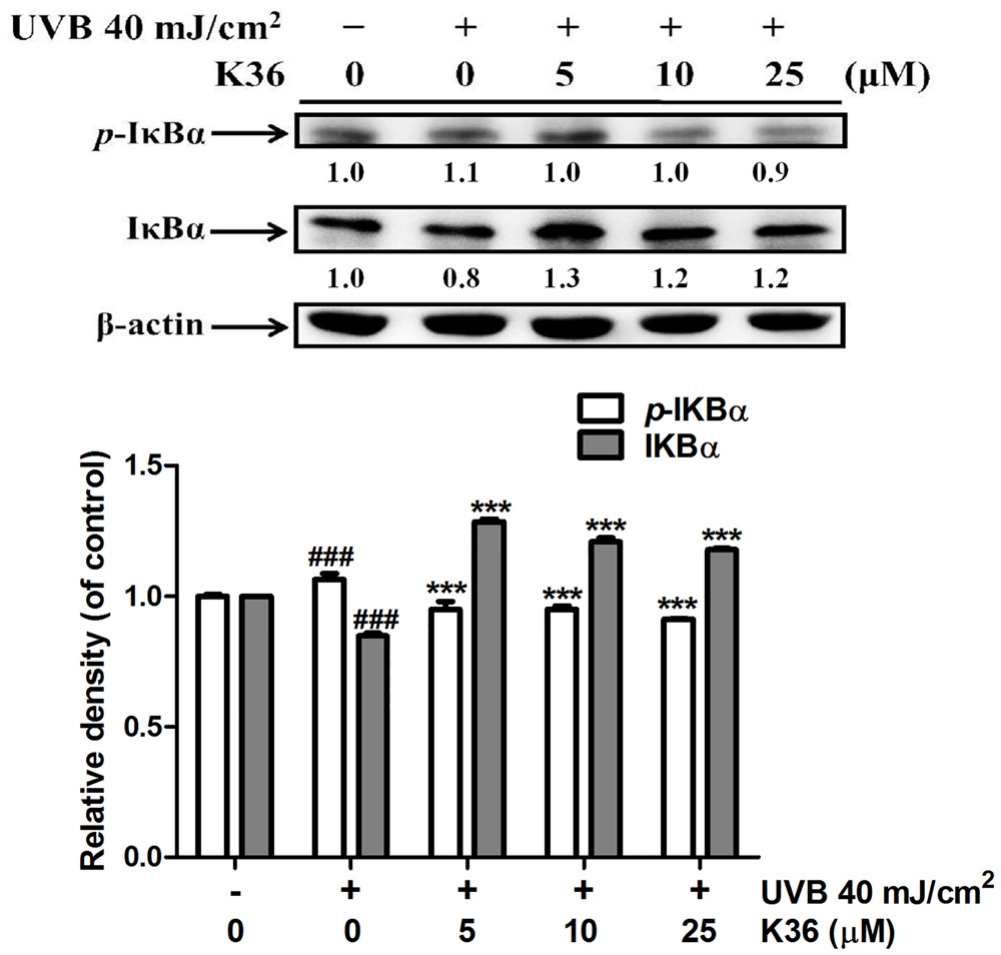
Effect of K36 on UVB-mediated *p*-IκBα and IκBα expression in human skin fibroblasts. Significant difference versus control group: #, *P* < 0.05; ##, *P* < 0.01. Significant difference versus nontreatment group: *, *P* < 0.05; **, *P* < 0.01; ***, *P* < 0.001.

**Fig 4:**
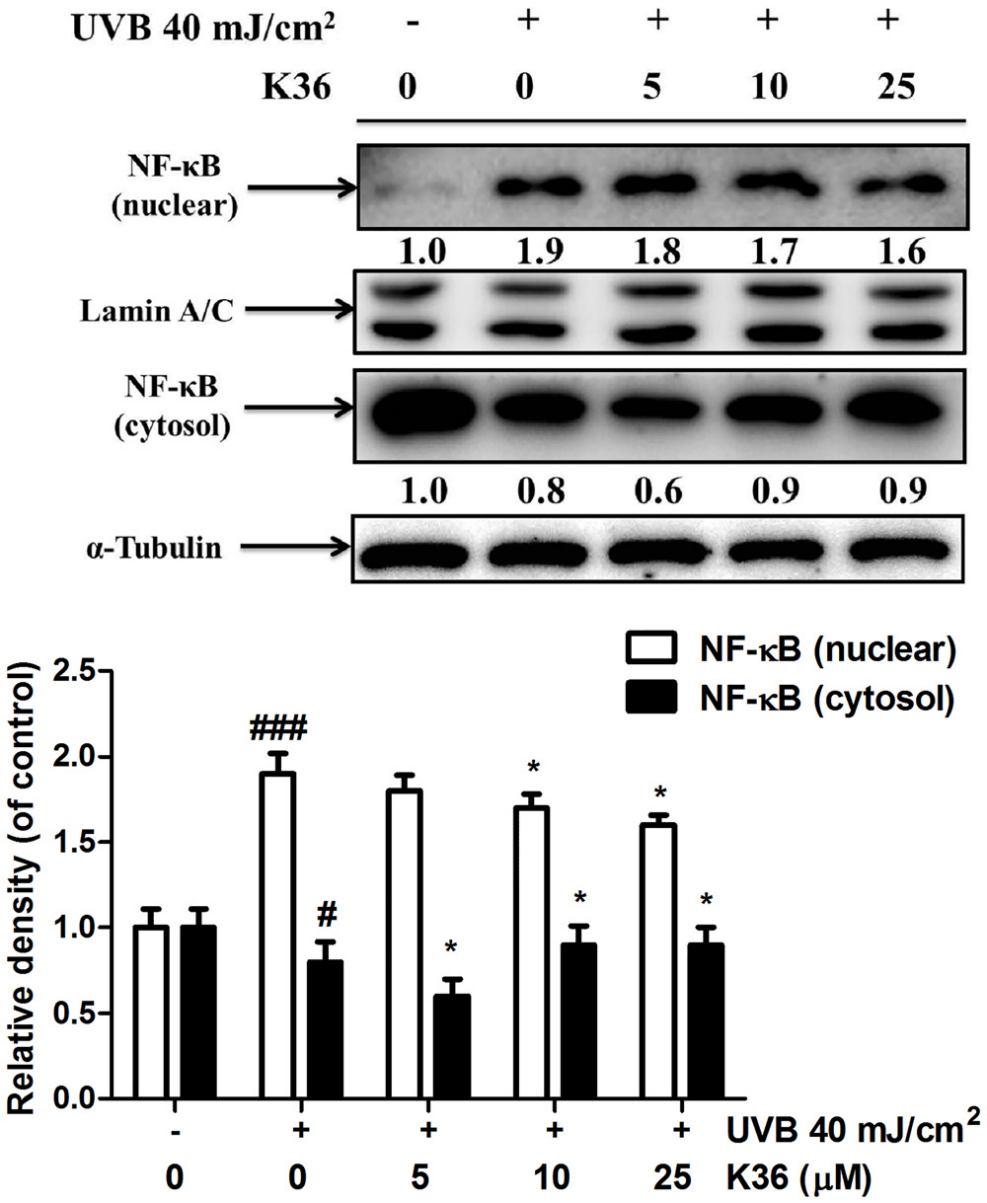
Effect of K36 on UVB-mediated NF-κB expression in nucleus of human skin fibroblasts.

1. **Effect of K36 on iNOS expression**. K36 treatment affected iNOS expression in fibroblasts, and K36 dose-dependently inhibited UVB-induced iNOS expression (Fig 1). iNOS expression increased 1.2-fold after UVB irradiation; however, K36 treatment at 5 μM significantly reduced iNOS levels to 0.7-fold compared with that of the control. In addition, the iNOS expresession was 0.5-fold of control after 25 μM K36 treatment. The iNOS expression was reduced after K36 treatment in a dose-dependent manner.
2. **Effect of K36 on COX-2 expression**. Compared with the COX-2 levels in the control cells, COX-2 levels were 1.5-fold higher in the UVB-irradiated fibroblasts (Fig 1). K36 treatment (5, 10, and 25 μM) reduced UVB-induced COX-2 expression; this effect was significant (decrease in magnitude from 1.5-fold to 0.7-fold compared with that of the control cells) when the dose exceeded 5 μM (Fig 1).
3. **Effect of K36 on COX-2 expression following treatment with MAP kinase inhibitors**. COX-2 expression is regulated by MAP kinases. Following treatment with PD98059 (ERK inhibitor), the JNK inhibitor II, and SB203580 (p38 inhibitor), COX-2 expression decreased. Cotreatment with K36 and the inhibitors, particularly the JNK inhibitor II and SB203580, further reduced COX-2 expression (Fig 2). These results indicated that K36 inhibited UVB-induced COX-2 overexpression through MAP kinase inhibition.
4. **Effect of K36 on the IκB/NF-κB pathway**. The results of western blotting indicated that UVB irradiation inhibited IκBα expression through ubiquitination and elevated *p*-IκBα expression. Nuclear translocation of NF-κB caused an inflammatory response. Compared with the control cells, UVB irradiation elevated *p*-IκBα expression by 1.1-fold, and 25 μM K36 significantly reduced *p*-IκBα expression (Fig 3). However, 5 μM K36 significantly increased IκBα expression (from 0.8-fold to 1.3-fold compared with the control cells) (Fig 3). As shown in Fig 4, UVB-induced the translocation of NF-κB into nucleus and the protein expression of NF-κB was 1.9-fold of control. After 25 μM K36 treatment, the protein expression of NF-κB was reduced to 1.6-fold of control in the nucleus.

**Fig 5:**
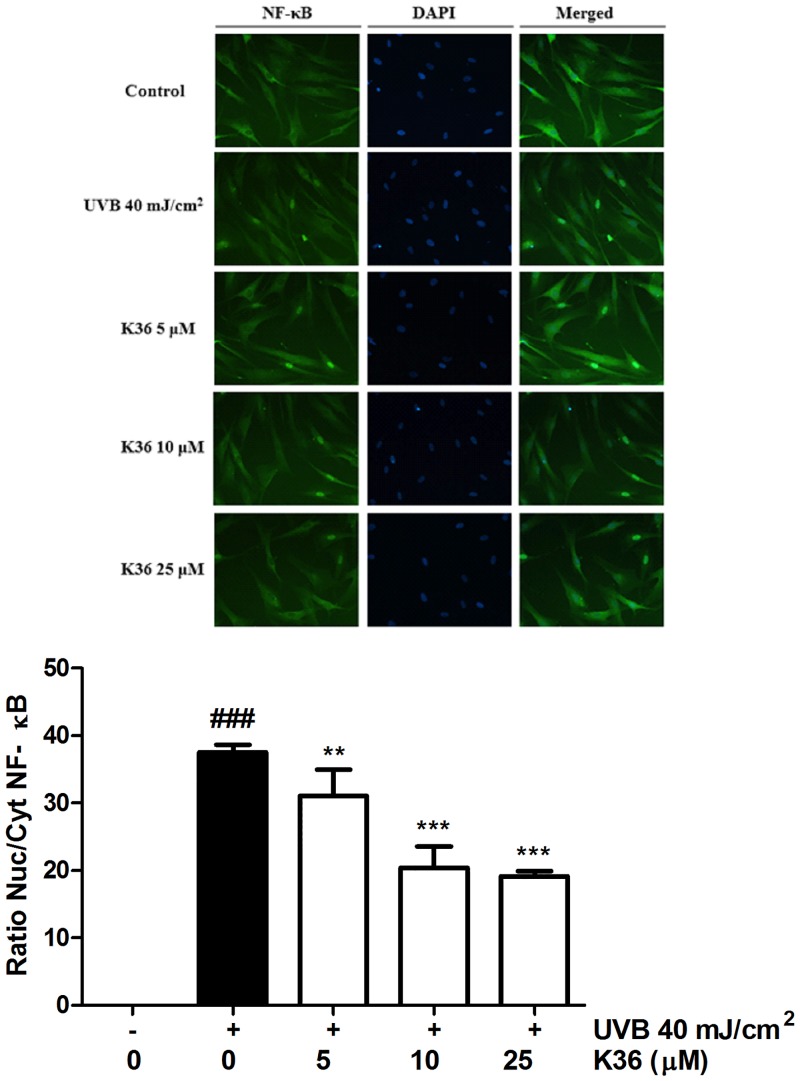
Effect of K36 on UVB-induced translocation of NF-κB (p65) in human fibroblasts.

Immunofluorescence staining of NF-κB was performed to determine the level of NF-κB activation in fibroblast cells. UVB irradiation increased the nuclear translocation of NF-κB, whereas K36 treatment inhibited this effect (Fig 5). The results of immunofluorescence staining for the NF-κB were consistent with protein expression western blotting. According to the results, K36 prevented UVB-induced inflammation through IκB/NF-κB pathway.

### Topical application of K36 on UVB-irradiated mouse skin

**Fig 6:**
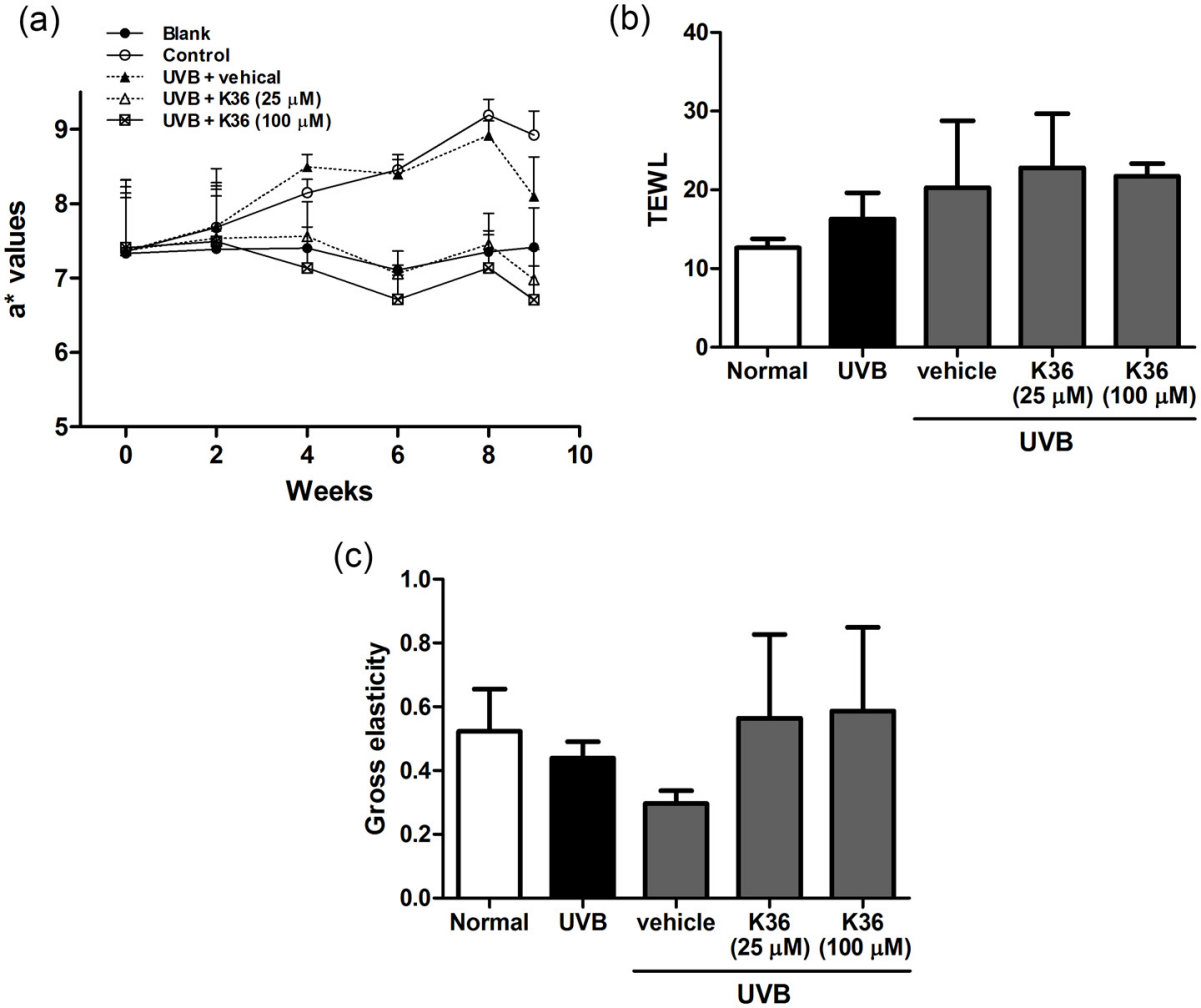
(a) Effect of K36 on a* values in chronic UVB-irradiated hairless mice. (b) Effect of K36 on TEWL in UVB-irradiated hairless mice in the tenth week. (c) Effect of K36 on total skin elasticity in chronic UVB-irradiated hairless mice in the tenth week.

1. **Effect of K36 on protection of skin from UVB-induced skin erythema and damage**. The body weights of mice in the five groups were not significantly different (data not shown). Erythema of the skin (a* value) indicates the degree of inflammation. In the present study, UVB irradiation induced skin inflammation; the a* values increased in the second week and significantly increased in the fourth week (Fig 6a). K36 treatment reduced erythema; however, the a* values for the 100-μM-K36-treated mice were similar to those for the control mice. The results indicated that K36 inhibited UVB-induced skin erythema and inflammation. These results were consisting to the inhibition of K36 on UUB-induced inflammatory in human skin fibroblasts.

UVB exposure increased transepidermal water loss (TEWL) in mice. After 10 weeks of K36 application to the hairless mice, no significant differences in TEWL were observed between the control and UV-irradiated groups (Fig 6b). The results indicated that K36 did not cause skin damage or toxicity.

2 **Measurement of skin elasticity and wrinkles induced by UVB irradiation**. Compared with skin elasticity in the control mice, skin elasticity in the UVB-irradiated mice decreased after 10 weeks of UVB exposure. Compared with the vehicle-treated and UVB-irradiated mice, the K36-treated mice showed inhibition of the decrease in UVB-irradiation-induced skin elasticity (Fig 6c).

**Fig 7:**
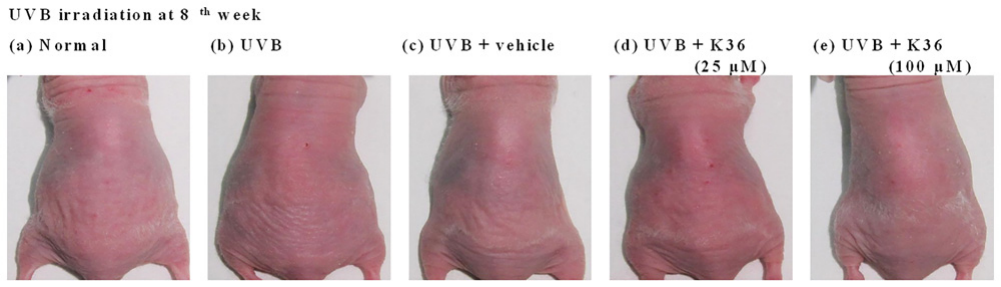
Photographs of wrinkles induced by UVB irradiation and the effect of topically applied K36.

The degree of wrinkle formation was assessed from the photograph of each mouse according to the grading scale described by Kim et al. [21], and the name of the group was unrevealed to the researcher. Wrinkle formation was observed macroscopically in the dorsal region following the initiation of UVB irradiation (Fig 7 and Table 1). Topically applying K36 (25 and 100 μM) reduced wrinkle formation (Fig 7); the wrinkle score was 4.5 ± 1.3 for UVB-irradiated mice, and the score significantly decreased to 2.6 ± 1.8 and 0.8 ± 1.0 after 25 and 100 μM K36 treatments, respectively (Table 1).

**Table 1:** Effect of K36 on skin wrinkles induced by UVB irradiation in hairless mice.

	Wrinkle score (8 weeks)
Normal mice	0.9 ± 1.0^a^
UVB-irradiated mice	4.5 ± 1.3^b^
Vehicle-treated UVB-irradiated mice	2.7 ± 2.1^c^
K36 (25 μM)-treated UVB-irradiated mice	2.6 ± 1.8^c^
K36 (100 μM)-treated UVB-irradiated mice	0.8 ± 1.0^a^

Values not followed by a common letter are significantly different (*p* < 0.05).

**Fig 8:**
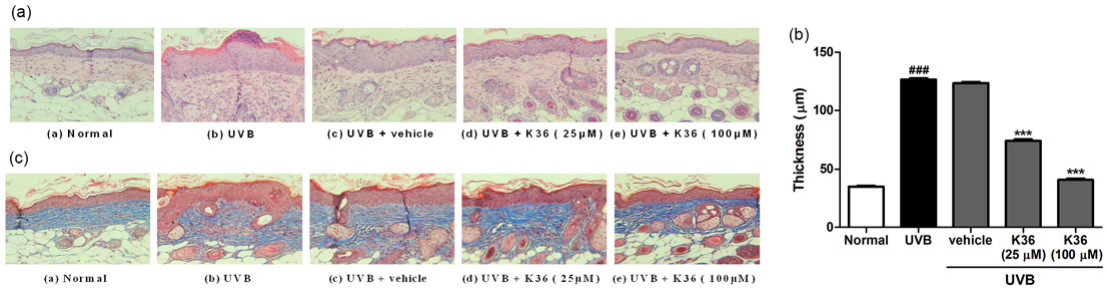
(a) Light micrographs of histological sections stained with hematoxylin and eosin in hairless mice. (b) The thickness of epidermis in hairless mice. (c) Light micrographs of histological sections stained with Masson's trichrome in hairless mice. Collagen fibers were stained in blue.

3 **Effects of K36 on the thickness of the epidermis and collagen in UVB-irradiated hairless mice skin**. Histological examination was performed to understand the effects of K36 on the thickness, lesion formation, and amount of collagen in the skin following UVB irradiation. Upon UVB exposure, the skin thickness of the UVB-irradiated mice increased significantly compared with that of the control mice (Fig 8a). Topically applying K36 significantly inhibited the increase in epidermal thickness induced by UVB exposure (Fig 8a). The thickness of epidermis was 35.1 ± 0.6 μm in control group, and 126.5 ± 1.3 μm after UVB irradiation (Fig 8b). After K36 treatment, the thickness of epidermis was 373.8 ± 1.4 μm at 25 μM K36 group and 41.0 ± 0.9 μm at 100 μM K36 group (Fig 8b). The results indicated that K36 ameliorated UVB-induced hyperplasia of epidermis.

Histological observation of the dorsal skin by using Masson's trichrome staining indicated that the collagen content in the UVB-irradiated mice was significantly lower than that in the control mice. K36 treatment restored the collagen content in the dermis of the hairless mice (Fig 8c).

### *In vitro* transdermal delivery of K36

To evaluate the potential of K36 for clinical application, the present study assessed the efficiency of percutaneous absorption. The time-dependent cumulative absorption of K36 was measured using the Franz vertical diffusion cell assembly. The flux of K36 was approximately 0.41 ± 0.23 nmol/cm^2^/h, and the skin deposition was 0.09 ± 0.02 nmol. These results indicated that K36 can penetrate and remain in the skin of hairless mice.

## Discussion

The results of present study indicated that K36 inhibited UVB-induced iNOS and COX-2 expression in human skin fibroblasts. In addition, K36 regulated IκBα/*p*-IκBα and NF-κB expression and inhibited the nuclear translocation of NF-κB in human skin fibroblasts. K36 inhibited the increase in skin thickness and wrinkle formation, and the reduction in skin elasticity induced by long-term UVB irradiation in the hairless mice. Furthermore, immunohistochemical staining results indicated that topically applying K36 inhibited the degradation of collagen in the skin of the hairless mice.

UV exposure increases activator protein-1 (AP-1) expression, a transcription factor that upregulates the gene and protein expression of MMPs [23]. MMPs, especially MMP-1, MMP-3 and MMP-9, degrade the ECM (e.g., collagen and elastin) causing coarse wrinkles and sagging skin [18, 24, 25]. In our previous study, K36 inhibited the MMP/AP-1 expression causing an increase in collagen content in human skin fibroblasts [14]. Therefore, it seems likely that the protective effect of K36 on skin photoaging induced by chronic UVB exposure may be attributed to the increase in collagen synthesis, the inhibition of MMP expression in dermal fibroblasts, and the inhibition of epidermal hyperplasia.

Acute exposure to UV irradiation causes characteristic inflammation signs such as erythema. UV-induced inflammation is mediated by COX-2 and iNOS [10]. UV-induced ROS drive the activation of MAP kinases (i.e., ERK, JNK, and p38), recruiting AP-1 (c-Fos and c-Jun) to the nucleus and subsequently activating NF-κB and upregulating proinflammatory gene expression [26, 27]. A study reported that MAP kinases and AP-1 regulated COX-2 expression [26]. UV irradiation was reported to cause nuclear translocation of NF-κB, thus inducing MMP production for degrading collagen in the human skin [28, 29]. In addition, NF-κB-modulated COX-2 and iNOS gene transcription and protein expression cause skin inflammation [9]. AP-1 and NF-κB modulate COX-2 and iNOS expression [26]. In this study, K36 inhibited UV-irradiation-induced iNOS and COX-2 overexpression, the expression of p-IκBα and NF-κB and the translocation of NF-κB. In addition, the inhibition of COX-2 expression by K36 was related to the regulation of the MAP kinase pathway in human skin fibroblasts. In an animal study, K36 reduced erythema (a* value), an index for inflammation. These results showed that K36 exhibited antiinflammatory activity in human skin fibroblasts and in hairless mouse skin and suggest that K36 protects the skin from UV-induced photodamage through its antiinflammatory activity.

Skin aging is manifested as an increase in skin thickness and wrinkle formation and a reduction in skin elasticity, which is fundamentally associated with reductions in the level of collagen type I, the principal component of the dermal layer of the skin [30]. Chronic UVB irradiation induces keratinocyte proliferation and epidermal hyperplasia and reduces the production of type I procollagen, thereby leading to the loss of collagen and, consequently, to increases in wrinkle formation and skin thickness as well as the reduction of skin elasticity [31]. In our previous study, K36 treatment inhibited the protein expression of UVB-irradiation-induced AP-1 and the phosphorylation of MAP kinases in fibroblasts, protecting the skin from photoaging [14]. In the present study, K36 protected mice skin from UVB-induced wrinkle formation and collagen degradation. Skin elasticity was related to the content of collagen and elastin, and K36 restored collagen content, thus potentially contributing to the elasticity of mouse skin. Thickening of the epidermis is a characteristic of skin photoaging. K36 reduced the thickness of epidermis and the loss of collagen and, hence, may contribute in preventing photodamage and photoaging.

## Conclusion

**Fig 9:**
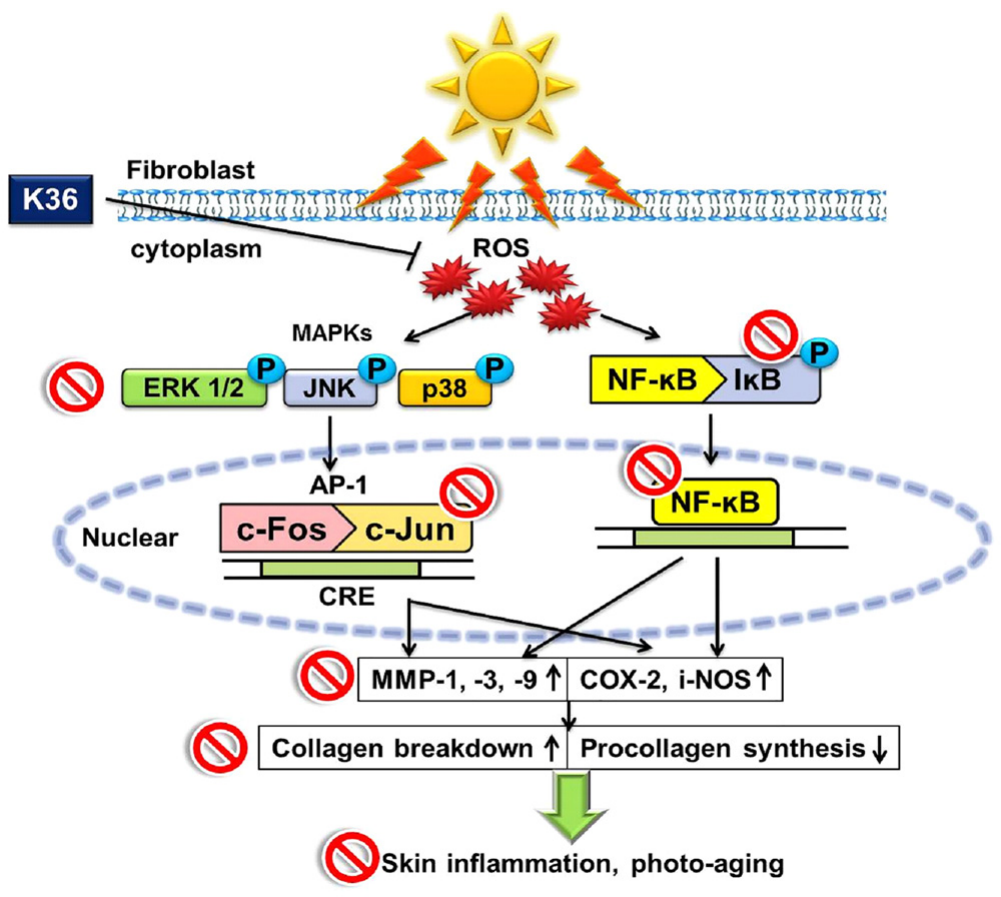
Schematic diagram showing inhibitory effects of K36 in UVB induced inflammatory and photodamage.

In this study, K36 reduced UVB-irradiation-induced iNOS and COX-2 expression, modulating the IκB/NFκB pathway (Fig 9). K36 reduced UVB-induced wrinkle formation, inflammation, skin thickening, and collagen degradation. In addition, K36 penetrated the skin of hairless mice. K36 can serve as an ideal antiaging and antiphotoaging agent, exhibiting potential for use in the therapeutic and cosmetic industries.
